# Progress and Prospects of CRISPR/Cas Systems in Insects and Other Arthropods

**DOI:** 10.3389/fphys.2017.00608

**Published:** 2017-09-06

**Authors:** Dan Sun, Zhaojiang Guo, Yong Liu, Youjun Zhang

**Affiliations:** ^1^Longping Branch, Graduate School of Hunan University Changsha, China; ^2^Department of Plant Protection, Institute of Vegetables and Flowers, Chinese Academy of Agricultural Sciences Beijing, China

**Keywords:** CRISPR/Cas9, insects, non-insect arthropods, research progress, prospects

## Abstract

Clustered regularly interspaced short palindromic repeats (CRISPR) and the CRISPR-associated gene Cas9 represent an invaluable system for the precise editing of genes in diverse species. The CRISPR/Cas9 system is an adaptive mechanism that enables bacteria and archaeal species to resist invading viruses and phages or plasmids. Compared with zinc finger nucleases and transcription activator-like effector nucleases, the CRISPR/Cas9 system has the advantage of requiring less time and effort. This efficient technology has been used in many species, including diverse arthropods that are relevant to agriculture, forestry, fisheries, and public health; however, there is no review that systematically summarizes its successful application in the editing of both insect and non-insect arthropod genomes. Thus, this paper seeks to provide a comprehensive and impartial overview of the progress of the CRISPR/Cas9 system in different arthropods, reviewing not only fundamental studies related to gene function exploration and experimental optimization but also applied studies in areas such as insect modification and pest control. In addition, we also describe the latest research advances regarding two novel CRISPR/Cas systems (CRISPR/Cpf1 and CRISPR/C2c2) and discuss their future prospects for becoming crucial technologies in arthropods.

## Introduction

**Figure 1:**
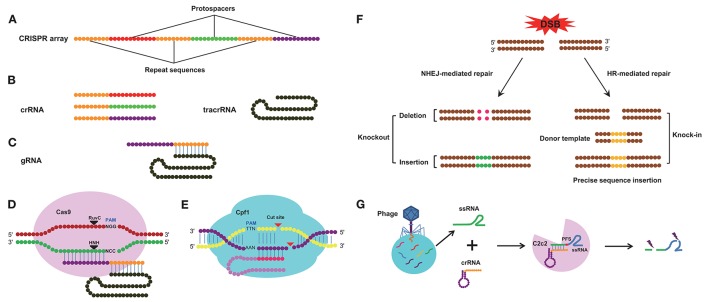
The architecture of the CRISPR/Cas system. **(A)** Diagram of the CRISPR array. CRISPR array consists of protospacers and repeat sequences. **(B)** Constituent elements of the crRNA transcribed from the whole CRISPR array and tracrRNA transcribed from repeat sequences of the CRISPR array. **(C)** Composition of the gRNA. The gRNA consists of crRNA and tracrRNA. **(D)** Schematic of cleavage by the Cas9 enzyme. The Cas9 enzyme recognizes PAM (NGG) site and cleaves target DNA sequence between the third and fourth bases near the PAM site. **(E)** Schematic of cleavage by the Cpf1 enzyme, the Cpf1 enzyme recognizes PAM (TTN) site and cleaves target DNA sequence independent of tracrRNA. **(F)** The repair pathway of double strand break (DSB) mediated by CRISPR system. The DSB induced by the Cas9/sgRNA complex can be repaired by non-homologous end joining (NHEJ) or homologous recombination (HR). This can result in small insertions or deletions at the target sites (left) and homologous repair with a desired template (right). This can be used to alter the genome by means of gene knockout and knock-in. **(G)** Schematic of cleavage by the C2c2 CRISPR effector. crRNA directs C2c2 enzyme to cleave single-stranded RNA (ssRNA) target sequences and the cleavage activity is dependent on the protospacer flanking site (PFS).

Genome editing technologies are useful for understanding the functions of target genes in diverse organisms (Segal and Meckler, 2013). Before the CRISPR/Cas9 system was discovered, zinc finger nucleases (ZFNs) and transcription activator-like effector nucleases (TALENs) technologies were used for genome modification; both technologies can be used to design a DNA-binding domain that can effectively recognize and modify virtually any sequence, and both technologies have been widely applied in various fields (Gaj et al., 2013). ZFNs and TALENs, however, require the use of a variety of nucleases, and the off-target effects of nucleases can lead to cellular toxicity. In addition, methods using ZFNs and TALENs are complex and labor-intensive (Kanchiswamy et al., 2016). These two genome-editing systems have been recently replaced by the CRISPR/Cas9 system, which is far more convenient and effective than ZFNs and TALENs (Lander, 2016; Mohanraju et al., 2016; Wang H. et al., 2016; Westra et al., 2016). Compared with RNA interference (RNAi) technology, CRISPR/Cas9 generates changes at the genomic level that are stable and heritable, and the mutant gene can be transmitted to the next generation, while gene silencing by RNA interference is usually an instantaneous process, unless the dsRNA is supplied continuously (Perkin et al., 2016). CRISPR loci are typically composed of a clustered set of CRISPR-associated (Cas) genes and a signature CRISPR array, which includes a series of repeat and spacer sequences (Hsu et al., 2014). The spacer sequences (protospacers) are variable and originate from invading DNA; these spacer sequences combine with the repeat sequences to form the CRISPR-RNA (crRNA), and each crRNA hybridizes with a *trans*-activating crRNA (tracrRNA) to form a single guide RNA (sgRNA) (Deltcheva et al., 2011) (Figures 1A–C). The sgRNA then combines with the Cas9 nuclease (Jiang et al., 2013) and directs Cas9 to cleave complementary target DNA sequences adjacent to a protospacer-adjacent motif (PAM), typically the sequence NGG (where N represents any base), thereby creating a double-strand break (DSB) in the DNA sequence (Jinek et al., 2012). However, not all foreign DNA is incorporated into CRISPR arrays. Previous studies have confirmed that the CRISPR system favors spacers generated from free DNA ends, narrowing its choice of DNA sequences (Modell et al., 2017). The Cas9 nuclease-induced DSBs can be repaired via two major approaches, non-homologous end-joining (NHEJ) and homology-directed repair (HDR) (Sander and Joung, 2014), and these distinct DSB repair mechanisms can be further subdivided depending on the nature of the generated DNA ends (Wyman and Kanaar, 2006). NHEJ will give rise to insertion or deletion (Indel) mutations that disrupt the open reading frame (ORF) of target genes, by this way, we can realize knockout of target DNA sequence and HDR can be used to introduce specific mutations or insert sequences of interest in accordance with the invading DNA template by homologous recombination (HR), in this way, we can realized knock-in of specific gene (Figure 1F). The Cas9 nuclease is derived from *Streptococcus pyogenes* (Ceasar et al., 2016) and contains two active sites, the resistance to ultraviolet C (RuvC) endonuclease site at the amino-terminal end and the (histidine-asparagine-histidine, H-N-H) HNH endonuclease site in the middle of the protein, both of which can cleave exogenous double-stranded DNA (dsDNA) (Figure 1D), the HNH nuclease domain cleaves the DNA strand that is complementary to the crRNA; the RuvC-like nuclease domain of Cas9 cleaves the DNA strand opposite the complementary strand (Doudna and Charpentier, 2014). Recently, an improved class 2 CRISPR/Cas system known as the CRISPR/Cpf1 system has been described; in this system, the Cpf1 enzyme from *Acidaminococcus* and *Lachnospiraceae* can mediate robust genome editing in human cells (Zetsche et al., 2015). Cpf1 is smaller than the standard Cas9 and is easily delivered to cells and tissues; furthermore, a single RNA is sufficient to guide Cpf1 cleavage. Cpf1 cuts dsDNA in a different manner than Cas9, creating a distal staggered cut near a 5′ PAM (TTN) site and leaving an overhang at the exposed end (Figure 1E). In addition to the CRISPR/Cpf1 system, another CRISPR/Cas system called CRISPR/C2c2 has been developed. In this system, a single-RNA-guided CRISPR effector, C2c2, from *Leptotrichia shahii* can manipulate single-stranded RNA (ssRNA) target sequences (Figure 1G), but its ssRNA cleavage activity is dependent on the nucleotide 3′ adjacent to the target site, known as the protospacer flanking site (PFS). Although Cas9, Cpf1, and C2c2 are all able to edit target sequences in the CRISPR/Cas system, there are many differences in several aspects among these three CRISPR/Cas systems (Table 1).

**Table 1:** A comparison of Cas9, Cpf1, and C2c2 in the CRISPR system.

	**Cas9**	**Cpf1**	**C2c2**
Target type^a^	dsDNA	dsDNA	ssRNA
Single guide RNA^b^	crRNA-tracrRNA	crRNA	crRNA
Cleavage recognition^c^	NGG	TTN	PFS
Spacer length^d^	20 bp	>17 bp	28 bp
Cleavage type^e^	Blunt-end cleavage	Staggered-end cleavage	cleaves ssRNA
Active cleavage site^f^	RuvC and HNH	RuvC	HEPN
Cleavage site inactivation^g^	DNA nickase	cannot cleave	cannot cleave

a *The type of Cas9, Cpf1, and C2c2 enzymes cleave target sequence. dsDNA, double strands DNA; ssRNA, single-stranded RNA*.

b *Single guide RNA (sgRNA) directs Cas9, Cpf1, and C2c2 enzyme to cleave target sequence, in this process, Cas9 depends on the complex of crRNA-tracrRNA, while Cpf1 and C2c2 only need crRNA*.

c *The recognition sites of Cas9, Cpf1, and C2c2. Among them, Cas9 recognizes NGG, Cpf1 recognizes TTN (N is any base), C2c2 depends on PFS site*.

d *The length of sgRNA*.

e *The end of target DNA or RNA cleaved by Cas9, Cpf1, and C2c2 enzymes*.

f *The cleavage domains of Cas9, Cpf1, and C2c2 enzymes. Among them, the cleavage domains of Cas9 are RuvC and HNH, the cleavage domains of Cpf1 are two RuvC-like domains, and the cleavage sites of C2c2 are two HEPN-like domains*.

g *If the cleavage site is inactivated, Cas9 formed DNA nickase can still cut dsDNA, however, Cpf1 and C2c2 can't cleave target sequence*.

This year marks the tenth anniversary of the identification of the biological function of CRISPR/Cas as adaptive immune systems in bacteria (Barrangou and Horvath, 2017). In just a decade, CRISPR/Cas9 technology has been widely used to modify genome sequences in diverse species ranging from microbes and plants to animals and even humans. The majority of these studies were conducted in model organisms. Researchers first used CRISPR/Cas9 technology to introduce desired single- and multi-nucleotide mutations into the *Streptococcus pneumoniae* and *Escherichia coli* genomes (Jiang et al., 2013). Ronda et al. demonstrated that CrEdit (CRISPR/Cas9-mediated genome editing) combined with the EasyClone vector system could be used to manipulate the genomics DNA of *Saccharomyces cerevisiae* by integrating three genes into three different integration sites to produce β-carotene (Ronda et al., 2015). In 2013, scientists introduced the *S. pyogenes* Cas9 (SpCas9) and an artificial chimeric gRNA (chigRNA) into *Arabidopsis thaliana* and *Nicotiana benthamiana*, which was the first attempt to edit the genomes of model plants (Li et al., 2013). In recent years, researchers have exploited the CRISPR/Cas9 system by injecting sgRNA and Cas9 into zygotes to modify the genomes of many other organisms, such as cattle (Heo et al., 2015; Wang, 2015), goats (Ni et al., 2014), pigs (Hai et al., 2014; Lai et al., 2016; Zhou et al., 2016), rabbits (Honda et al., 2015), and malaria parasites (Singer and Frischknecht, 2017). Regarding arthropods, several reviews have summarized the successful application of different genome editing technologies, especially the CRISPR/Cas9 system, in insects (Beumer and Carroll, 2014; Xu et al., 2015; Chen et al., 2016; Reid and O'Brochta, 2016; Venken et al., 2016; Cui et al., 2017; Taning et al., 2017); however, there is still no comprehensive review that covers both insect and non-insect arthropods. Meanwhile, more recently, the rapid development of omics and molecular biology has resulted in the increased use of CRISPR/Cas9 technology in both insect and non-insect arthropods, and the results of these studies are not summarized in the reviews mentioned above. In this review, we have elaborated on the application and prospects of the CRISPR/Cas system in insects of groups including Diptera, Lepidoptera, Coleoptera, and Orthoptera and non-insect arthropods of the subclass Acarina and the groups Decapoda, Amphipod, and Diplostraca. Undoubtedly, CRISPR/Cas9 technology will facilitate the exploration of gene functions and provide promising new strategies for field control of both insect and non-insect arthropods. Hence, summarizing the use of CRISPR/Cas9 technology in arthropods is of great significance for the sustainable development of global agriculture, forestry, and fisheries and is relevant to public health.

## Application of CRISPR/Cas9 in arthropods

**Figure 2:**
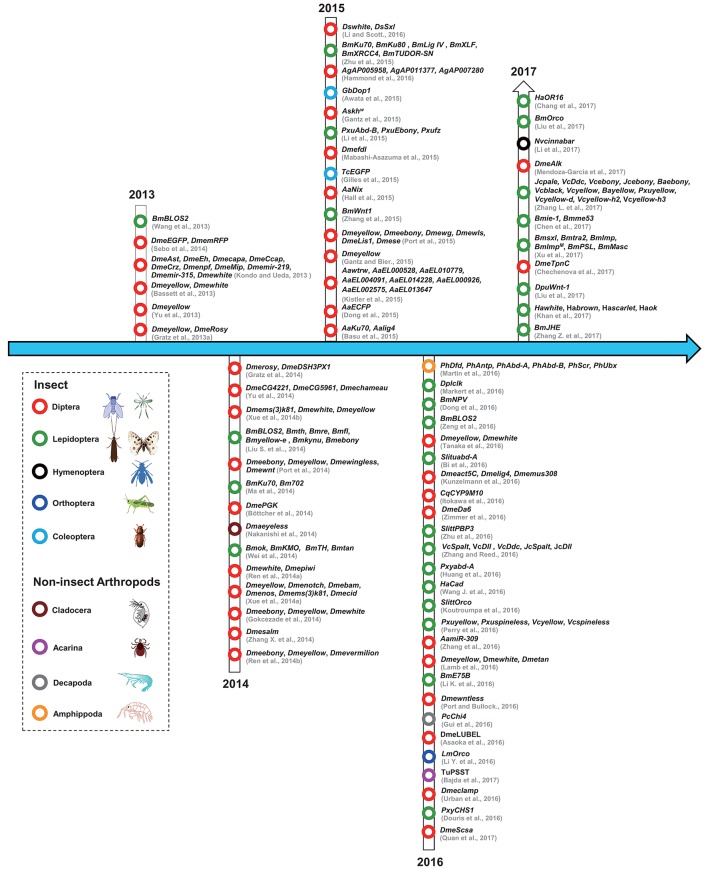
The history of the use of CRISPR/Cas9 technology in arthropods. The dotted lines indicate different arthropod classifications. Red circles represent Diptera; black circles represent Hymenoptera; dark blue circles represent Orthoptera; cambridge blue circles represent Coleoptera; gray circle represents Decapoda; green circles represent Lepidoptera; dark red circles represent Cladocera; purple circle represents Acarina; orange circle represents Amphippoda. The citations in this figure are rigorously sorted out according to their accepted dates of the relevant journals. Lepidoptera (Bm, *Bombyx mori*; Ha, *Helicoverpa armigera*; Dpl, *Danaus plexippus*; Dpu, *Dendrolimus punctatus*; Vc, *Vanessa cardui*; Jc, *Junonia coenia*; Ba, *Bicyclus anynana*; Slitt, *Spodoptera littoralis*; Slitu, *Spodoptera litura*; Pm, *Papilio machaon*; Pxu, *Papilio xuthus*; Pxy, *Plutella xylostella*); Coleoptera (Tc, *Tribolium castaneum*; Gb, *Gryllus bimaculatus*); Diptera (Dme, *Drosophila melanogaster*; Ds, *Drosophila suzukii*; Cq, *Culex quinquefasciatus*; As, *Anopheles stephensi*; Ag, *Anopheles gambiae*; Aa, *Aedes aegypti*); Orthoptera (Lm, *Locusta migratoria*); Hymenoptera (Nv, *Nasonia vitripennis*); Acarina (Tu, *Tetranychus urticae*); Decapoda (Pc, *Palaemon carinicauda*); Cladocera (Dma, *Daphnia magna*); Amphipoda (Ph, *Parhyale hawaiensis*). For interpretation of the references to color in this figure legend, the reader is referred to the web version of this article.

The studies that have used CRISPR/Cas9 in insect and non-insect arthropods have been summarized according to the year of publication and the species involved (Figure 2). From the timeline, we can see that the first use of the CRISPR/Cas9 system in arthropods was in the model insect *Drosophila melanogaster*, followed by *Bombyx mori*; afterwards, the system was used in diverse non-model insects including different types of flies, mosquitos, moths, butterflies, and other non-insect arthropods. The detailed information has also been organized to facilitate a comparison of these studies (Table 2).

**Table 2:** The application of the CRISPR/Cas9 system in insects and non-insect arthropods.

**Classification**	**Species**	**Genes**	**Mutation**	**Delivery**	**Summary**	**References**
Diptera	*Drosophila melanogaster*	*yellow, rosy*	Knockout, Knock-in	mRNA	The CRISPR/Cas9 system was first used to mediate efficient genome engineering in *Drosophila*.	Gratz et al., 2013b
		*yellow, white*	Knockout	mRNA	Knockout of the *yellow* gene was highly efficient and concentration dependent, and different sgRNAs had different editing efficiencies.	Bassett et al., 2013
		*yellow*	Knockout	mRNA	Targeted multiple genes with different sgRNAs and achieved remarkably effective targeted mutagenesis.	Yu et al., 2013
		*Ast, Eh, capa, Ccap, Crz, npf, Mip, mir-219, mir-315, white*	Knockout	Transgene	Crossed two transgene vectors that harbored Cas9 and an sgRNA to obtain a Cas9-sgRNA complex.	Kondo and Ueda, 2013
		*rosy, DSH3PX1*	Knockout, Knock-in	Plasmid, transgene	Performed efficient and complex genomic manipulations using CRISPR/Cas9-mediated HDR.	Gratz et al., 2014
		*ebony, yellow, wingless, wnt*	Knockout, Knock-in	Transgene	Different promoters had different abilities to drive sgRNA expression.	Port et al., 2014
		*EGFP, mRFP*	Knockout	Plasmid, transgene	Induced mutations by injecting an sgRNA into Vasa-Cas9 transgenic fly embryos.	Sebo et al., 2014
		*white, piwi*	Knockout, Knock-in	Plasmid, transgene	The use of Cas9 nickase and sgRNA pairs can prevent off-target effects when generating indel mutants.	Ren et al., 2014a
		*ms(3)k81, white, yellow*	Knockout, Knock-in	Plasmid, transgene	Transgenic Cas9 efficiently induced gene knock-in or knockout in *Drosophila*.	Xue et al., 2014a
		*yellow, notch, bam, nos, ms(3)k81, cid*	Knockout	Plasmid, transgene	A CRISPR/Cas9-mediated conditional mutagenesis system combined with tissue-specific expression of Cas9 was used to temporally and spatially inhibit gene expression.	Xue et al., 2014b
		*salm*	Knock-in	mRNA, transgene	Proposed a two-step method to flexibly modify the fly genome.	Zhang X. et al., 2014
		*ebony, yellow, vermilion*	Knockout, Knock-in	Plasmid, transgene	Donor template and sgRNA plasmids were injected into Cas9 transgenic embryos in *Drosophila*.	Ren et al., 2014b
		*ebony, yellow, white*	Knockout, Knock-in	Plasmid, transgene	Construction of a bicistronic Cas9/sgRNA vector increased the efficiency of gene targeting.	Gokcezade et al., 2014
		*ebony, yellow, wg, wls, Lis1, Se*	Knockout, Knock-in	Plasmid, transgene	Transgenic individuals exhibited more efficient knock-in than non-transgenic individuals.	Port et al., 2015
		*yellow*	Knock-in	Transgene	Converted heterozygous recessive mutations to homozygous loss-of function mutations using mutagenic chain reaction (MCR) technology in *Drosophila*.	Gantz and Bier, 2015
		*Dα6*	Knock-in	Plasmid	The G275E mutation of the nAChR *Dα6* subunit is directly related to spinosad resistance.	Zimmer et al., 2016
		LUBEL	Knockout	Plasmid, transgene	Flies with LUBEL mutations exhibited reduced survival and defective climbing in response to heat.	Asaoka et al., 2016
		*Scsα*	Knockout	Plasmid	Mutant flies could not produce sufficient energy to promote normal growth.	Quan et al., 2017
Diptera	*Drosophila melanogaster*	*clamp*	Knockout	Plasmid, transgene	An essential transcription factor regulated the expression of a sex-specific gene.	Urban et al., 2016
		Phosphoglycerate kinase	Knockout	Plasmid	CRISPR/Cas9 technology was combined with overlap PCR-based donors to achieve chromosomal gene manipulation in S2 cells.	Böttcher et al., 2014
		*chameau, CG4221, CG5961*	Knock-in	mRNA	Tested the efficiency of HDR-mediated genome modifications and resolved a problem associated with “ends-in” recombination.	Yu et al., 2014
		*fdl*	Knockout	Plasmid	CRISPR/Cas9 can be used to analyze or manipulate protein glycosylation pathways.	Mabashi-Asazuma et al., 2015
		*mod(mdg4)*	Knockout	Plasmid, transgene	Validated a functional gene involved in trans-splicing that affected development in flies.	Gao et al., 2015
		*act5C, lig4, mus308*	Knockout, Knock-in	Plasmid	Provided a comprehensive toolbox for genome editing in *Drosophila* S2 cells.	Kunzelmann et al., 2016
		*yellow, white, tan*	Knock-in	Plasmid	Proposed a new method of achieving single or multiple allelic substitutions in regions of interest.	Lamb et al., 2016
		*wntless*	Knockout	Plasmid	A tRNA-sgRNA complex can increase the cleavage efficiency of the Cas9 and Cpf1 nucleases.	Port and Bullock, 2016
		*TpnC*	Knockout	Plasmid	Verified that the *TpnC* gene is related to myofibril assembly.	Chechenova et al., 2017
		*Alk*	Knockout	Plasmid, transgene	Demonstrated that transcription factors can influence *Alk* gene expression by introducing mutations in *Alk* enhancer regions.	Mendoza-Garcia et al., 2017
	*Drosophila suzukii*	*white, Sxl*	Knockout	Plasmid	*Sxl* is an excellent gene for validating the CRISPR/Cas9 system and could be used to suppress populations of this destructive pest.	Li and Scott, 2016
	*Drosophila subobscura*	*yellow, white*	Knockout	mRNA	Demonstrated the analysis of gene functions in a non-model *Drosophila* species.	Tanaka et al., 2016
	*Aedes aegypti*	*miR-309*	Knockout	mRNA, Cas9 protein	Knocking out the miR-309 gene resulted in severe ovarian defects.	Zhang et al., 2016
		*ECFP*	Knockout	mRNA	The CRISPR/Cas9 system was used to introduce mutations in *A. aegypti* for the first time.	Dong et al., 2015
		*Nix*	Knockout	mRNA	Provided a new vector mosquito management strategy by converting female mosquitoes into harmless male individuals.	Hall et al., 2015
		*wtrw, AaEL000528, AaEL010779, AaEL004091, AaEL014228, AaEL000926, AaEL002575, AaEL013647*	Knockout, Knock-in	mRNA, Cas9 protein, plasmid	Generated different types of mutations using diverse repair mechanisms.	Kistler et al., 2015
		*Ku70, lig4*	Knockout, Knock-in	mRNA, Cas9 protein	A transient embryo assay was used to rapidly identify highly effective sgRNAs, promoting gene editing efficiency.	Basu et al., 2015
Diptera	*Anopheles stephensi*	*kh^w^*	Knock-in	Plasmid	The CRISPR/Cas9 system was combined with MCR to increase HDR-mediated germ-line gene conversion.	Gantz et al., 2015
	*Culex quinquefasciatus*	*CYP9M10*	Knockout	mRNA	Demonstrated that the *CYP9M10* gene is associated with pyrethroid resistance.	Itokawa et al., 2016
	*Anopheles gambiae*	*AgAP005958, AgAP011377, AgAP007280*	Knockout, Knock-in	Plasmid	Targeted female reproduction-related genes to control mosquito populations.	Hammond et al., 2016
Lepidoptera	*Spodoptera litura*	*Slabd-A*	Knockout	mRNA	*Slabd-A*-deficient individuals exhibited defects in body segmentation and pigmentation.	Bi et al., 2016
		*SlitPBP3*	Knockout	mRNA	Showed that the *SlitPBP3* gene plays an essential role in the perception of female sex pheromones.	Zhu et al., 2016
	*Spodoptera littoralis*	*SlitOrco*	Knockout	mRNA	Investigated the function of the *Orco* gene in the non-model insect *Spodoptera littoralis*.	Koutroumpa et al., 2016
	*Helicoverpa armigera*	*HaCad*	Knockout	mRNA	Demonstrated that the *HaCad* gene is related to Bt toxin Cry1Ac resistance using genome editing technology.	Wang J. et al., 2016
		*white, brown scarlet, ok*	Knockout	mRNA	Essential genes interacted with non-essential genes to produce diverse insect pigmentation patterns.	Khan et al., 2017
		*OR16*	Knockout	Plasmid, mRNA	Provided a novel strategies to destroy pest insect mating.	Chang et al., 2017
	*Plutella xylostella*	*Pxabd-A*	Knockout	mRNA	CRISPR/Cas9 was used to target genes in *Plutella xylostella* for the first time, providing novel ideas for pest management.	Huang et al., 2016
		*PxCHS1*	Knockout	Plasmid	Described the resistance management strategies of major agricultural pests and elucidated the resistance mechanism mode of action using the CRISPR/Cas9 system.	Douris et al., 2016
	*Dendrolimus punctatus*	*DpWnt-1*	Knockout	mRNA	Indicated that the *DpWnt-1* signaling pathway is necessary for anterior segmentation and appendage development.	Liu H. et al., 2017
	*Bombyx mori*	*BmBLOS2*	Knockout	mRNA	Used the defective phenotypic effects caused by gene mutations to verify the functionality of the CRISPR/Cas9 genome editing system.	Wang et al., 2013
		*Bm-ok, BmKMO, BmTH, Bmtan*	Knockout	mRNA	Validated the CRISPR/Cas9 technology and assessed mutation efficiency using defective phenotypic effects.	Wei et al., 2014
		*BmBLOS2, Bmth, Bmre, Bmfl, Bmyellow-e, Bmkynu, Bmebony*	Knockout	Plasmid	Verified that the CRISPR/Cas9 system induced multiple gene mutations simultaneously in *B. mori*.	Liu Y. et al., 2014
		*BmKu70, Bm702*	Knockout	Plasmid	Proved that individuals lacking *BmKu70*, a component of the NHEJ pathway, exhibit increased homologous recombination (HR)	Ma et al., 2014
		*BmWnt1*	Knockout	mRNA	Exploited a new method to study the *Wnt1* signaling pathway in *B. mori*.	Zhang et al., 2015
Lepidoptera	*Bombyx mori*	*BmKu70, BmKu80, BmLigIV, BmXLF, BmXRCC4, BmTUDOR-SN*	Knockout, Knock-in	Plasmid	Demonstrated that the lack of NHEJ-related factors could increase HR activity using the CRISPR/Cas9 system.	Zhu et al., 2015
		*BmNPV*	Knockout	Plasmid	Constructed a virus-induced CRISPR/Cas9 system and achieved antiviral responses in *B. mori*.	Dong et al., 2016
		*Bmie-1, Bmme53*	Knockout	Plasmid, transgene	Large-segment deletions of the *BmNPV* gene mediated by transgenic CRISPR/Cas9 were used as an antiviral therapy.	Chen et al., 2017
		*BmBLOS2*	Knockout	Plasmid	U6-driven sgRNAs beginning with four different nucleotides could cause single site mutations in *B. mori*.	Zeng et al., 2016
		*Bmsxl, Bmtra2, Bmlmp, BmPSL Bmlmp^*M*^, BmMasc*	Knockout	Plasmid	Verified that these six genes are related to sex determination.	Xu et al., 2017
		*BmJHE*	Knockout	Plasmid	Showed that *BmJHE* regulates life span and is associated with the insulin/TOR pathway.	Zhang Z. et al., 2017
		*E75B*	Knockout	mRNA	The 20E primary response gene *E75B* could regulate steroidogenesis.	Li K. et al., 2016
	*Junonia coenia*	*Spal, Dll*	Knockout	mRNA, Cas9 protein	These two genes are involved in the formation and development of eyespot patterns.	Zhang and Reed, 2016
	*Vanessa cardui*					
	*Papilio machaon*	*Abd-B*	Knockout	mRNA	Mutant individuals had four pairs of extra prolegs, which were not observed in wild-type individuals.	Li X. Y. et al., 2016
	*Danaus plexippus*	*clk*	Knockout	mRNA	Defined the role of the *clk* gene in the control of migration behavior.	Markert et al., 2016
	*Papilio xuthus Vanessa cardui*	*yellow, spineless*	Knockout	mRNA, Cas9 protein	Verified the three-way stochastic choices that expand color vision in butterflies.	Perry et al., 2016
	*Papilio xuthus Papilio machaon*	*Abd-B, ebony, fz*	Knockout	mRNA	Provided a valuable genomic and genetic technology for studying butterflies and other insects.	Li et al., 2015
	*Junonia coeniaPapilio machaon Bicyclus anynana*	*pale, Ddc, ebony, black, yellow, yellow-d, yellow-h2, yellow-h3*	Knockout	mRNA, Cas9 protein	Identified candidate genes by comparative RNA-Seq and verified the function of these candidate genes, which are related to melanin pigmentation, using CRISPR/Cas9.	Zhang L. et al., 2017
Orthoptera	*Locusta migratoria*	*Orco*	Knockout	mRNA	Generated loss-of-function mutants for functional genetic studies of locusts and for managing insect pests.	Li Y. et al., 2016
Coleoptera	*Gryllus bimaculatus*	*Dop1*	Knockout	mRNA	Dopamine and octopamine neurons mediated aversive and appetitive reinforcement, respectively, in crickets.	Awata et al., 2015
	*Tribolium castaneum*	*EGFP*	Knockout Knock-in	Plasmid, mRNA	CRISPR/Cas9 technology was used to edit the *Tribolium* genome for the first time and to achieve the knockout or knock-in of a target gene.	Gilles et al., 2015
Hymenoptera	*Nasonia vitripennis*	*cinnabar*	Knockout	mRNA, Cas9 protein	Established CRISPR/Cas9-directed gene editing in *N. vitripennis*.	Li et al., 2017
Acarina	*Tetranychus urticae*	*PSST*	Knockout	Plasmid, transgene	Demonstrated that an H92R amino acid substitution in the PSST homolog was related to pyridaben resistance and introduced the mutation into the *Drosophila PSST* homolog using CRISPR/Cas9 genome editing tools.	Bajda et al., 2017
Decapoda	*Palaemon carinicauda*	*EcChi4*	Knockout	mRNA	A shrimp/decapod genome was successfully edited for the first time.	Gui et al., 2016
Amphipod	*Parhyale hawaiensis*	*Dfd, Antp, Abd-A, Abd-B, Scr, Ubx*	Knockout	mRNA	Described the function of these six Hox genes in crustaceans and indicated the phenotypic effects caused by different Hox gene changes.	Martin et al., 2016
Cladocera	*Daphnia magna*	*eyeless*	Knockout	mRNA	CRISPR technology was used to edit the *D. magna* genome for the first time and produced a deformed eye phenotype.	Nakanishi et al., 2014

### Diptera

#### Drosophila

As a model insect, *D. melanogaster* is at the forefront of genetic analysis, and the application of CRISPR/Cas9 technology in *Drosophila* has promoted the use of genome editing technology in other insects (Bassett and Liu, 2014). In 2013, Gratz et al. first used CRISPR/Cas9 technology to introduce mutations into the *Drosophila* genome, generating a 4.6 kb deletion in the *yellow* locus by utilizing two target sgRNAs and a single-stranded oligonucleotide donor (ssODN) template (Gratz et al., 2013a). Furthermore, they discussed the potential applications of CRISPR/Cas9-mediated genome editing technology and described the benefits of generating designer flies on demand (Gratz et al., 2013b). In the same year, researchers described a method of increasing the frequency of HR using a reintegration vector (Baena-Lopez et al., 2013). Yu et al. compared the efficiencies of TALEN- and CRISPR/Cas9-mediated HDR mechanisms, constructed an easy-to-screen platform, and developed three HDR approaches for precise mutagenesis (Yu et al., 2014). Researchers have reported the construction of two transgene vectors expressing the Cas9 protein and an sgRNA and the subsequent crossing of flies carrying these transgenes to obtain an active sgRNA-Cas9 complex in the germline as well as the injection of an sgRNA-encoding plasmid into transgenic-Cas9 flies, which allowed the knockout or knock-in of different target genes (Kondo and Ueda, 2013; Gratz et al., 2014; Port et al., 2014; Ren et al., 2014a; Sebo et al., 2014; Xue et al., 2014a,b). Three methods have been used to induce HDR in flies; several researchers injected donor template and sgRNA plasmids into transgenic Cas9 embryos (Gratz et al., 2014; Ren et al., 2014b; Zhang X. et al., 2014), while Port et al. injected a donor template plasmid into transgenic embryos containing Cas9 and an sgRNA (Port et al., 2014), and Gokcezade et al. injected donor template, Cas9 and sgRNA plasmids into non-transgenic individuals (Gokcezade et al., 2014). Subsequently, the Port group compared these three methods of facilitating HDR using the same gRNA and donor plasmid and found that the use of transgenic individuals produced a higher knock-in frequency than non-transgenic individuals (Port et al., 2015).

Scientists are constantly looking for new fly research methods, which has undoubtedly accelerated the development of this model organism as well as other *Drosophila* species. Several studies have reported the use of genome editing techniques to introduce mutations in *Drosophila* cell lines (Böttcher et al., 2014; Gao et al., 2015; Lin et al., 2015; Mabashi-Asazuma et al., 2015; Kunzelmann et al., 2016), and a GFP-expressing cell line and comprehensive workflow were established for analyzing functional *Drosophila* genes. Thus, research at the cellular level will facilitate the optimization of the gene editing system and the establishment of a simple and convenient platform (Kunzelmann et al., 2016). In 2015, Gantz et al. developed a mutagenic chain reaction (MCR) method that could produce autocatalytic mutations and used this method to convert heterozygous mutations to homozygous mutations (Gantz and Bier, 2015). The CRISPR/Cas9 system was used to introduce a site-specific mutation (G275E) into the nicotinic acetylcholine receptor (nAChR) *D*α*6* subunit, and although the spinosad insecticide resistance level of flies with the G275E mutation (66-fold higher than non-mutated flies) was lower than that of individuals with a *D*α*6*-null mutation (311-fold higher than non-mutated flies), it sufficiently demonstrated that the G275E mutation is directly related to spinosad resistance (Zimmer et al., 2016). Another study reported the development of a method for scarless allele replacement in *Drosophila*, which was shown to be suitable for single or multiple allelic substitutions in regions of interest (Lamb et al., 2016).

Some studies focused on specific genes and the defective phenotypes observed when these genes were knocked out using CRISPR/Cas9 technology. Bassett et al. targeted the *yellow* gene using the CRISPR/Cas9 system and found that the system was highly efficient and concentration dependent, as the efficiency of gene editing increased with increasing concentrations of the sgRNA; however, the adult survival rate was reduced when higher sgRNA concentrations were used (Bassett et al., 2013). Yu et al. also targeted the *yellow* gene and demonstrated greatly increased efficiency (Yu et al., 2013). Based on the use of CRISPR/Cas9 technology in *D. melanogaster*, researchers introduced site-specific mutations into the *white* (*w*) and *Sex lethal* (*Sxl*) genes of *Drosophila suzukii*. The mutant phenotype of white eyes was generated at a low efficiency, which may have occurred because the flies were injected with plasmid DNA encoding Cas9 and the sgRNA other than mRNA, in contrast to mRNA, the plasmid DNA needs to experience a transcription process *in vivo*. In addition, it may also has been due to the specificity of the *white* gene and *Drosophila* species. Mutation of the *Sxl* gene resulted in abnormal genitalia and reproductive tissues in female individuals (Li and Scott, 2016). Another study revealed the critical role of succinyl-CoA synthetase/ligase (SCS), which is associated with metabolites in *Drosophila*. The authors generated a mutation in the SCS alpha subunit (*Scs*α) using the CRISPR/Cas9 system and observed that *Scs*α-deficient individuals exhibited developmental delays, impaired locomotor activity and increased mortality under starvation conditions (Quan et al., 2017). Thus, as stated above, *Scs*α is essential for proper energy metabolism in *Drosophila*. Asaoka et al. utilized the CRISPR/Cas9 system to establish linear ubiquitin E3 ligase (LUBEL)-deficient flies, which exhibited reduced survival and defective climbing in response to heat (Asaoka et al., 2016). A sex-specific gene has also been studied in *Drosophila* using CRISPR/Cas9. To ensure that the transcriptional activity of the male X chromosome is equal to that of the two female X chromosomes, the male-specific lethal (MSL) complex is recruited to regions of the X chromosome; this process is dependent on the chromatin-linked adapter for MSL proteins (CLAMP) zinc finger protein. Urban et al. used the CRISPR/Cas9 system to generate mutations in the *clamp* gene in flies and found that *clamp*-null males and females died at different developmental stages and that the expression of a transcription factor was altered in a sex-specific manner (Urban et al., 2016). Also in *Drosophila*, the troponin C (TpnC) gene was shown to be associated with muscle formation using CRISPR/Cas9 technology (Chechenova et al., 2017). Typically, mutations in the coding region of a gene are studied; however, a recent paper reported that CRISPR/Cas9 technology could be used to mutant transcription factor binding sites in enhancer regions of the *Alk* locus in *Drosophila*, thereby mediating the expression of the *Alk* gene (Mendoza-Garcia et al., 2017).

#### Aedes aegypti, anopheles stephensi, culex quinquefasciatus and Anopheles gambiae

Mosquitoes can spread many diseases that pose a serious threat to human health, such as Zika, malaria, dengue, chikungunya, and filariasis (Gabrieli et al., 2014; Reegan et al., 2017). For the past several decades, synthetic insecticides have been used to control vector mosquitoes. Not only do these insecticides affect the ecological environment (Bayen, 2012), they also induce resistance in vector mosquitoes (Tikar et al., 2009). As a result of their blood-triggered reproductive strategy, female mosquitoes must feed on blood before mating with male individuals, and they then lay their eggs in water. A female mosquito can lay eggs 3–4 times in her life, producing 150–250 individuals each time. Understanding this breeding mechanism can help us to control the mosquito population. *Aedes aegypti* is a vector of many human-borne arboviruses, such as yellow fever, dengue, and chikungunya (Weaver and Barrett, 2004). Targeting mosquito genes of interest *in vivo* using a genome editing tool has become popular, and ZFNs and TALENs have been successfully used in mosquitoes (Aryan et al., 2013; Smidler et al., 2013). In 2015, scientists first used CRISPR/Cas9 technology to modify the genome of *A. aegypti* (Dong et al., 2015), targeting the ECFP gene in transgenic *A. aegypti* mosquitoes harboring DsRed and ECFP marker genes and obtaining individuals that expressed DsRed but not ECFP. Kistler et al. injected optimal sgRNA-Cas9 mixtures into *A. aegypti* embryos and demonstrated that CRISPR/Cas9 could induce different mutations using diverse repair mechanisms (Kistler et al., 2015). Basu et al. established a platform for the rapid screening of candidate sgRNAs using a transient embryo assay, which enhanced the efficiency of gene editing and improved HR-mediated repair rates (Basu et al., 2015).

Non-coding microRNAs can regulate a series of physiological processes, including ecdysis, metamorphosis, embryogenesis, and host-pathogen interactions (Lucas et al., 2013, 2015b), and are regarded as a new frontier in mosquito biology. miRNA-1174 is responsible for sugar and blood uptake in *A. aegypti* (Liu S. et al., 2014). microRNA-8 is expressed in the fat body and can regulate the *A. aegypti* reproductive process (Lucas et al., 2015a). The functional verification of a microRNA is usually performed using an RNA interference technique. A recent study targeted microRNA-309 using the CRISPR/Cas9 system, resulting in a severe loss of ovarian function, growth retardation, substantially decreased follicle numbers, and a reduced egg hatching rate in mutant *A. aegypti* individuals. Additionally, the authors identified that the homeobox 4 (*SIX4*) gene was a direct target of miR-309 (Zhang et al., 2016). These phenotypic defects were consistent with the effects caused by antagomir silencing (a novel class of chemically engineered oligonucleotides) which can silence the expression of endogenous gene, but the results mediated by CRISPR/Cas9 were more efficient and clear. Because only female mosquitoes feed on blood and transmit pathogens (Papathanos et al., 2009), the conversion of female mosquitoes into harmless male individuals has shown promise as a new vector management strategy. Male determination in *A. aegypti* is controlled by the M factor, which is a dominant male-determining factor located on the Y chromosome in the M locus. Hall et al. targeted an M factor functional gene, *Nix*, in *A. aegypti* using the CRISPR/Cas9 system, which led to the feminization of *Nix*^−^ males (Hall et al., 2015). In *Anopheles gambiae*, which is the major vector of malaria, researchers utilized CRISPR/Cas9 technology to target three genes related to a female-sterility phenotype (Hammond et al., 2016). In *Anopheles stephensi*, Gantz et al. established an efficient autonomous CRISPR/Cas9-mediated gene drive system originating from the MCR method and demonstrated that the progeny of males and females derived from transgenic males exhibited a high frequency of germ-line gene conversion, likely as a result of HDR (Gantz et al., 2015). The CRISPR/Cas9 method has also been used to reveal the relationship between detoxifying enzymes and insecticide resistance in *Culex quinquefasciatus* (Itokawa et al., 2016); when the cytochrome P450 gene *CYP9M10* was targeted in a resistant strain of *C. quinquefasciatus*, the individuals carrying no functional *CYP9M10* copy exhibited an ~110-fold reduction in permethrin resistance, demonstrating that *CYP9M10* is a crucial factor associated with pyrethroid resistance. Overall, these studies show that the CRISPR/Cas9-mediated genome editing system can be used to effectively modify the mosquito genome and potentially to control the population of mosquitoes and disease transmission. The biosafety risks of this technology related to the release of CRISPR/Cas9-edited insects into the environment should be considered when selecting the most suitable, environmentally friendly method of implementing the gene driven system (Esvelt et al., 2014; Oye et al., 2014; Alphey, 2016; Champer et al., 2016; Taning et al., 2017).

### Lepidoptera

Following the launch and achievement of the silkworm genome project (Xia et al., 2004), the silkworm genome database has propelled the progress of functional genomics research in *B. mori* (Xia et al., 2014). Recently, the CRISPR/Cas9 technology has also provided a new tool to analyze functional genes in diverse insects, and *B. mori* is the first agricultural insect achieving efficient CRISPR/Cas9 genome editing of functional genes. In Lepidoptera, except for the model insect *B. mori*, there are plenty of major agricultural pests affecting crop yields, and some of them can have crop-devastating effects. Furthermore, the frequent and incorrect use of traditional chemical pesticides has led to high resistance in many of these major lepidopteran insects including *Plutella xylostella, Helicoverpa armigera, Spodoptera littoralis*, and *Spodoptera litura*, which makes them more difficult to control. Fortunately, over the past few years, CRISPR/Cas9-mediated target gene editing technology has been used in many insects, providing a new, environmentally friendly option for field pest control. Addtionally, butterflies in Nymphalidae, Danaidae, and Papilionidae are good model lepidopteran insects for insect genetics study, and CRISPR/Cas9-mediated genome editing tool has also be used to analyze the formation of eyespot wings and insect behaviors in these butterflies.

#### Bombyx mori

The lepidopteran insect *B. mori* is an important, economically valuable species as well as a model organism in scientific research. It is one of the earliest insect models in which a genomic editing tool was used to verify a gene function *in vivo*. Researchers compared three different genomic engineering techniques (ZFNs, TALENs, and CRISPR/Cas9) (Daimon et al., 2014) and found that the most convenient and effective method was the CRISPR/Cas9 system. The *BmBLOS2* gene was targeted to verify the application of CRISPR/Cas9 in *B. mori*; when this gene is mutated, the larval integument is changed from opaque to translucent (Wang et al., 2013; Liu Y. et al., 2014). Generally, mutation strategies cause small indels in a coding sequence; however, Liu et al. have reported the use of a method that can induce 3 kb fragment variations in non-coding sequences using a CRISPR system with two sgRNAs in the *B. mori* cell line BmNs, they designed two 20-bp sgRNAs adjacent to the PAM site with the typical sequence NGG (where N represents any base), the 3 kb fragment locates between both sgRNAs. They also designed six sgRNAs to target six different genes and cotransfected these six sgRNAs and Cas9 into BmNs cell line. Ultimately, the targeted mutations were detected for all target loci, indicating that CRISPR/Cas system can induce multiple genes mutations simultaneously. Therefore, the CRISPR/Cas9 system enables the production of large-fragment or multiple-gene mutations simultaneously in *B. mori* (Liu Y. et al., 2014; Ma et al., 2014). In another study, the authors took advantage of a notable mutant phenotype of the *Bm-ok* gene, which allowed homozygous mutants to be easily screened and the mutation efficiency of CRISPR/Cas9 technology to be calculated (Wei et al., 2014).

The *Wnt1* signaling pathway is important for embryonic development in insects. According to previous reports, the *Wnt1* gene is responsible for the formation of spot patterns (Yamaguchi et al., 2013) and normal abdominal segments in *B. mori* (Yamaguchi et al., 2011). The CRISPR/Cas9 system provides an ideal strategy for studying the *Wnt1* signaling pathway. Zhang et al. obtained *BmWnt1* gene knockout individuals and observed that the mutants exhibited defects in body segmentation and pigmentation in a dose-dependent manner and that the Hox homologous genes were down-regulated in *BmWnt1*-deficient individuals (Zhang et al., 2015). Studies have shown that the Fem piRNA is the primary female determining factor in *B. mori*. Xu et al. used a transgenic-based CRISPR/Cas9 system to elucidate the sex determination mechanism of male individuals. They generated a series of mutations in the *BmSxl, Bmtra2, Bmlmp, BmlmpM, BmPSI*, and *BmMasc* genes and studied individuals with each mutation, ultimately determining that the *BmPSI* gene is a critical auxiliary factor in determining the male sex in *B. mori* (Xu et al., 2017). Liu et al. indicated that deletion of *BmOrco* gene by utilizing CRISPR/Cas9 technology severely disrupts the olfactory system, furthermore, the homozygous mutant was unable to respond to sex pheromones (Liu Q. et al., 2017). Many reports have shown that the CRISPR/Cas9 system can be used to integrate donor DNA into the *B. mori* genome, but this process depends on several factors. A recent study indicated that the lack of NHEJ-related factors, such as *BmKu70, BmKu80, BmLigIV, BmXLF*, and *BmXRCC4*, increased HR efficiency mediated by CRISPR/Cas9 in *B. mori* (Ma et al., 2014; Zhu et al., 2015).

Several studies have successfully used CRISPR/Cas9 technology in gene therapy by disrupting a viral gene, such as HIV-1 or hepatitis B genes (Ebina et al., 2013; Seeger and Sohn, 2014; Liao et al., 2015; Pellagatti et al., 2015). However, there is currently no method that can completely eliminating a viral genome in insects. *B. mori* is the only host of *BmNPV*, which was exploited by scientists to construct a virus-induced CRISPR/Cas9 system, with the Cas9 nuclease being activated upon infection with the virus (Dong et al., 2016). This new system inhibited viral proliferation within a short time, and, importantly, the optimized system provided new ideas regarding the establishment of a virus-free transgenic line in *B. mori*. In addition, researchers targeted the *BmNPV ie-1* and *me53* genes in another study and successfully introduced mutations into these two genes; this method not only contributes to modern sericulture but also opens up options for future anti-viral therapies (Chen et al., 2017). Increasing numbers of functional genes have been identified by researchers using the CRISPR/Cas9 system in *B. mori*. Juvenile hormone (JH) and 20-hydroxyecdysone (20E) are responsible for insect growth and development. A loss-of-function analysis of *B. mori* JH esterase (*BmJHE*) was performed using a CRISPR/Cas9 transgenic system, and the mutant individuals exhibited an extended life span; this information could be used to extend the larval stage of *B. mori* to increase silk production (Zhang Z. et al., 2017). Furthermore, in another study, it was demonstrated that the *E75* isoforms of the 20E primary response gene mediated the regulation of steroidogenesis and developmental timing in *B. mori*. Of the three isoforms of *E75, E75A/C* is a positive transcriptional activator and induces the biosynthesis of ecdysteroid. Interestingly, scientists demonstrated that *E75B* had an opposite effect using CRISPR/Cas9 technology, as the synthesis of ecdysteroid was increased in *B. mori E75B* mutants (Li K. et al., 2016).

#### Spodoptera litura, spodoptera littoralis, and Plutella xylostella

*S. litura* is an omnivorous and destructive pest that is found throughout the world. Abdominal-A (*abd-A*) is an important gene for development in insects, and, using RNA interference in *B. mori*, scientists demonstrated that *Bmabd-A* is primarily responsible for normal development of the third to sixth body segments (Pan et al., 2009). Another study showed that as a transcription factor, *Bmabd-A* interacts with *BmPOUM2* during larva-to-pupa metamorphosis (Deng et al., 2012). Based on the axial patterning of the *Drosophila* cardiac tube, the *Ubx* gene is expressed in the aorta, and the homeotic genes *abd-A* and *abd-B* are expressed in the heart, indicating the genetic and functional diversity of this organ (Ponzielli et al., 2002). Recently, in *S. litura*, scientists targeted the *Slabd-A* gene using the CRISPR/Cas9 system and obtained *Slabd-A*-deficient individuals, which exhibited abnormal body segmentation and anomalous pigmentation (Bi et al., 2016). The same gene, *abd-A*, was also knocked out in *P. xylostella* using CRISPR/Cas9; injecting eggs with 500 ng/μl Cas9 mRNA resulted in a higher mutation rate (91%) for the *Pxabd-A* gene in G0 than injecting 300 ng/μl Cas9 mRNA (Huang et al., 2016). Phenotype analysis indicated that the *Pxabd-A* gene plays an essential role in promoting segmentation and gonad development in *P. xylostella* (Huang et al., 2016). The function of this gene is analogous to that in other Lepidoptera insects. Because the mutation mechanisms of the *Pxabd-A* gene are relatively complex, it is difficult to analyze their effects, and there are many issues to be resolved, such as the lower mutation rate in G2 and other roles of the *Pxabd-A* gene in insects.

Chitin is a natural amino polysaccharide involved in the formation of the extracellular matrix. The synthesis of chitin is primarily confined to epithelial cells under the cuticle and within the gut giving rise to peritrophic matrices of arthropods, some other invertebrates (nematode egg shells and some protozoan cyst walls) and fungal septa (septa, spores, and cell walls). Chitin synthesis is a target of many insecticides, fungicides, and acaricides (Merzendorfer, 2013). Many arthropod pests pose a serious threat to human health and life, including *A. gambiae*, the main vector of malaria (Hammond et al., 2016), *A. aegypti* is a potent vector of dengue, yellow fever, and chikungunya viruses (Kistler et al., 2015). The diamondback moth, *P. xylostella*, is a global lepidopterous pest of brassicaceous vegetables and was the first pest to evolve field resistance to Bt insecticides (Guo et al., 2015); it later developed basic resistance to almost all chemical insecticides. There are many chitin synthesis inhibitors. Benzoylureas (BPUs), buprofezin, and etoxazole are commonly used and share a set of molecular modes of action (MoAs) by directly interacting with chitin synthase. Initially, the acaricide etoxazole was used to inhibit chitin biosynthesis in the two-spotted spider mite, *Tetranychus urticae*. A single non-synonymous SNP was found in the chitin synthase 1 gene (*CHS1*) at position 1017, where an isoleucine (I) was replaced with a phenylalanine (F), and scientists demonstrated that this single amino acid replacement is related to etoxazole resistance (Van Leeuwen et al., 2012). At the same position as the spider mite I1017F mutation, Douris et al. identified that a mutation (I1042M) in the *CHS1* gene of BPU-resistant *P. xylostella*. Similarly, this is an amino acid substitution of isoleucine (I) to methionine (M), and the authors introduced both substitution mutations (I1056M/F) of the *CHS1* gene into *Drosophila* using the CRISPR/Cas9 system combined with the HDR pathway. These mutations corresponded to I1042M and I1017F in *P. xylostella* and *T. urticae*, respectively. The *Drosophila* lines that were homozygous for these mutations (I1056M/F) were highly resistant to BPUs, etoxazole and buprofezin (Douris et al., 2016); therefore, the CRISPR/Cas9 system provided a method of verifying MoA-resistance mechanisms *in vivo* and identified mechanisms that were shared across species.

The olfactory system of insects is very sensitive and depends on odor binding proteins to identify a variety of odorant substances. Pheromone binding proteins are the most important type of odor binding protein (Vogt and Riddiford, 1981). To illustrate the function of the pheromone binding protein gene in *S. litura*, researchers first mutated the pheromone binding protein 3 gene (*SlitPBP3*) using CRISPR/Cas9 genome editing technology and obtained *SlitPBP3* mutant individuals. Compared with wild-type males, the mutant males exhibited a significantly decreased response to sex pheromone components (Zhu et al., 2016). CRISPR/Cas9 genome editing tools were also used to target the olfactory receptor co-receptor (*Orco*) gene in *Spodoptera littoralis*, and the knockout individuals failed to respond to plant odors and sex pheromones (Koutroumpa et al., 2016). Compared with RNA interference techniques that are not suitable for Lepidoptera insects, the CRISPR/Cas9 system provides a more in-depth and stable method for studying functional genes.

#### Helicoverpa armigera

*Bacillus thuringiensis* (Bt) insecticides and Bt transgenic crops are widely used for pest control (Bravo et al., 2011). However, the evolution of resistance to Bt toxins has threatened the long-term development of pest management. A cadherin-like receptor was identified as a receptor of the Bt Cry1A toxin in several Lepidoptera insects (Wu, 2014). The cadherin-related receptor interacts with the Cry1Ab protoxin, which facilitates proteolytic cleavage and forms a pre-pore oligomeric structure in *Manduca sexta* (Gómez et al., 2002). Genetic linkage, cell toxicity and RNA interference experiments have demonstrated that cadherin is involved in Cry1Ac resistance in several Lepidoptera insects. Wang et al. first targeted exon 9 of the cadherin gene by injecting a mixture of *Cas*9 mRNA and an sgRNA into *Helicoverpa armigera* eggs. Using this CRISPR/Cas9 genome manipulation system, they obtained *HaCad* gene mutant individuals, which exhibited 549-fold higher resistance to Cry1Ac compared with a control strain (Wang J. et al., 2016). This result provided direct functional evidence that *HaCad* is a key receptor for Cry1Ac and is related to Cry1Ac resistance. CRISPR/Cas9 was also used in *H. armigera* to mutate four pigment genes, *white, brown, scarlet*, and *ok*; these mutations caused various physiological phenotypes in *H. armigera* (Khan et al., 2017). Recently, Chang et al. have demonstrated that the antagonist-mediated optimization of mating time ensures maximum fecundity using CRISPR/Cas9 system in *H. armigera*, which provides a new strategy to destroy pest mating (Chang et al., 2017).

#### Dendrolimus punctatus

The pine caterpillar moth, *Dendrolimus punctatus*, is a devastating forest pest in China and Southeast Asia, inducing severe defoliation and reducing resin production. Researchers first induced mutations in the *Wnt-1* gene using the CRISPR/Cas9 system to precisely and efficiently modify gene expression in *D. punctatus*. Previous reports indicated that the *Wnt-1* gene is associated with segmentation and development. In this paper, multiple mutant phenotypes were observed, such as abnormal posterior segments, defective legs, and head deformation (Liu H. et al., 2017). Therefore, the genome editing technology can be used to manipulate the genome of *D. punctatus* and, importantly, may provide a management strategy for a major defoliator.

#### Junonia coenia, vanessa cardui, papilio machaon, danaus plexippus and Papilio xuthus

The eyespot wings of nymphalid butterflies are colorful and appealing. Initially, the eyespot was located on the ventral hindwing and subsequently moved to dorsal wing surfaces. This apparent evolutionary mechanism is closely related to the function of the eyespot, which plays an important role in predation, courtship and sexual dimorphism (Monteiro, 2015). The eyespot pattern is regulated by a series of homologous genes (such as *Spalt, North, Dll*, and *en*) that form a single origin of expression and regulatory network (Reed and Serfas, 2004; Oliver et al., 2012). CRISPR/Cas9 is useful for studying the eyespot color patterns of the nymphalid butterflies *Junonia coenia* and *Vanessa cardui*. *Spalt* and *Distal-less (Dll)* are transcription factor genes that have been shown to play essential roles in promoting and inhibiting the formation of eyespot development, respectively (Zhang and Reed, 2016). Perry and colleagues verified the three-way stochastic choices that expand butterfly color vision using the CRISPR/Cas9 system (Perry et al., 2016). Recently, CRISPR/Cas9 was used to modify the *Papilio machaon* genome. The authors targeted four *Abd-B* genes and injected sgRNA/Cas9 mixtures into freshly collected *P. machaon* eggs; the mutants exhibited four pairs of extra prolegs on segments A7-A9, which were not observed in wild-type individuals (Li X. Y. et al., 2016). Marker et al. also defined the critical function of the *clk* gene in controlling migration behavior using TALENs and CRISPR/Cas9 genome editing tools in *Danaus plexippus*. They reported that injecting fewer than 100 eggs is sufficient to recover mutant progeny and to generate monarch knockout lines in ~3 months (Markert et al., 2016). In 2016, scientists integrated comparative genomics and CRISPR/Cas9 technology in two highly heterozygous and closely related butterflies, *Papilio xuthus* and *P. machaon*. They demonstrated interesting evolution patterns in butterflies and knocked out three genes that produce obvious phenotypes, *Abd-B, ebony*, and *fz*, using CRISPR/Cas9 technology (Li et al., 2015). This research provided a valuable genomic and genetic technology for studying butterflies and other insects. Soon after, Zhang et al. utilized comparative RNA-Seq technology to identify eight candidate genes associated with melanin pigmentation in four butterfly species and then knocked out these candidate genes to verify their function using the CRISPR/Cas9 system (Zhang L. et al., 2017). Thus, the combination of comparative genomics and CRISPR/Cas9 technology will become a powerful tool for discovering novel genes and revealing the behavioral mechanisms of non-model organisms.

### Orthoptera

#### Locusta migratoria

Locusts are important agricultural pests, and in the early days of locust outbreaks, governments issued policies to eradicate locusts. The traditional eradication tactics rely on synthetic insecticides, which increase the financial and environmental costs. Scientists also consider locusts to be important insects at the molecular level. One study showed that developmental synchrony is the foundation of gregarious behavior, migration and copulation. A miRNA (miR-276) gene was found to facilitate the synchrony of egg development and to directly determine the density of locusts (He et al., 2016). The olfactory system plays an important role in insects. Chemical pheromone signals are first received by peripheral tissues and processed in antennal nerve tissues, finally, olfactory and other sensory organs integrate the signals in the brain to direct insect behaviors, such as foraging, feeding, mating, and spawning (Leal, 2013). To further study the functional genes of *Locusta migratoria in vivo*, researchers used the CRISPR/Cas9 technique to modify the locust genome using an sgRNA targeting the odorant receptor co-receptor (*Orco*) gene. By microinjecting Cas9-mRNA and Orco-sgRNA into locust eggs, the authors achieved highly efficient mutation rates in the *Orco* gene, and they also successfully established *Orco* homozygous and heterozygous mutant lines (Li Y. et al., 2016). This is the first time the CRISPR/Cas9 system was used in locusts for genome editing, and the results not only suggested new ideas for locust control but also provided guidelines for using the technology in other insects from the orthopteran clade such as crickets.

#### Gryllus bimaculatus

Learning and memory are important functions of the human brain. Arthropods have a complex “brain” (Strausfeld, 2012) that enables them to adapt to different external environments. The central mechanisms of learning and memory are hotspot issues in insect neurobiology. Many studies have shown that the insect "brain" possesses associative learning abilities. In mammals, dopamine neurons are thought to play an important role in mediating both appetitive and aversive reinforcement. In insects, octopamine has been shown to participate in appetitive reinforcement, while dopamine has been shown to mediate aversive reinforcement. Honeybees have a strong olfactory learning and memory ability. In 2007, scientists demonstrated that octopamine and dopamine promoted appetitive and aversive reinforcement, respectively, in olfactory-based associative learning (Vergoz et al., 2007). Contrary to this result in honeybees, scientists proposed that dopamine in mushroom bodies mediates not only appetitive reinforcement but also aversive reinforcement in *Drosophila* (Krashes et al., 2009; Liu et al., 2012). To resolve these contradictions, Awata et al. knocked out the type 1 dopamine receptor gene (*Dop1*) using CRISPR/Cas9 technology in crickets (*Gryllus bimaculatus*). *Dop1* gene knockout crickets were defective in aversive learning with sodium chloride punishment but not in appetitive learning with water or sucrose reward (Awata et al., 2015). This study illustrated that CRISPR/Cas9 system is valuable in studies of associative learning and can be used to knock out related genes in arthropods.

### Coleoptera

#### Tribolium castaneum

The red flour beetle, *Tribolium castaneum* (Coleoptera), is a major pest of stored agricultural products. Beetles feed on grains, such as maize, wheat, and rice, lowering the nutritional value of food. Additionally, their secretions contain the carcinogen benzoquinone. The advent of a *T. castaneum* genomic database (Tribolium Genome Sequencing Consortium, 2008; Kim et al., 2010) has prompted researchers to look for genetic-based methods of eradication instead of the traditional fumigation; these methods could improve pest management and reduce the damage to the environment. Some approaches have been reported, including transgenesis (Pavlopoulos et al., 2004; Berghammer et al., 2009), RNA interference technology (Gilles and Averof, 2014), heat shock-mediated mis-expression of genes (Schinko et al., 2012), the GAL4/UAS system (Schinko et al., 2010), and the targeting of compensation mechanisms that are activated after disruption (Perkin et al., 2017). Subsequently, a simpler and faster genome editing tool, the CRISPR/Cas9 system, was first used in *T. castaneum* (Gilles et al., 2015). In this paper, Gilles et al. demonstrated that mutations in the *E-cadherin* gene caused severe defects in dorsal closure; moreover, they also achieved efficient homology-directed knock-in via the CRISPR-mediated system. In a sense, the use of this genome editing system in different species lays the foundation for establishing a transgenic method based on the CRISPR/Cas9 system, which could shorten the developmental gap between model organisms and non-model creatures.

### Hymenoptera

#### Nasonia vitripennis

Hymenoptera is the third largest clade of insects after Coleoptera and Lepidoptera and includes bees, ants, wasps, sawflies (Davis et al., 2010). The vast majority of hymenopterans are beneficial pollinators or predatory natural enemies of pests. The parasitoid wasp, *Nasonia vitripennis*, is not only an important natural enemy of pests but is also an ideal experimental insect. Li et al. used the CRISPR/Cas9 genome editing system to target the eye pigmentation gene *cinnabar* in *N. vitripennis*. They injected sgRNA/Cas9 mixtures into collected eggs, and effective and heritable mutants were obtained (Li et al., 2017). These results demonstrated that the gene manipulation system can be utilized to study haplo-diploid sex determination, axis pattern formation and other biological behaviors in *N. vitripennis*.

### Acarina

#### Tetranychus urticae

*Tetranychus urticae* is a polyphagous mite that can carry and transmit virus and can travel by wind and water and on insects, birds, humans, animals, all kinds of farm tools and flower seedlings. Mitochondrial respiratory complex I plays a key role in the synthesis and transfer of ATP in *T. urticae*, and NADH: ubiquinone oxidoreductase is crucial for oxidative energy conversion (Wirth et al., 2016). Mitochondrial electron transport inhibitors (METIs) including quinazolines, pyrimidinamines, pyrazoles, and pyridazinones are widely used to block the quinone binding receptor of complex I. Because of their frequent utilization, resistance to METI-Is evolves quickly (Stumpf and Nauen, 2001; Van Pottelberge et al., 2009). Bajda et al. identified an H92R amino substitution in the PSST homolog of complex I that is associated with METI-I resistance in a resistant *T. urticae* strain (Bajda et al., 2017). Subsequently, introduced the H92R mutation into the *Drosophila* PSST homolog using the CRISPR/Cas9 genome editing tool, but failed to obtain homozygous mutant fly individuals, indicating that the H92A amino substitution is lethal in *Drosophila* but not in *T. urticae* and that the mechanism of lethality requires further study (Bajda et al., 2017).

### Decapoda

#### Palaemon carinicauda

*Palaemon carinicauda* (falsely called *Exopalaemon carinicauda* previously) is an economically important shrimp that belongs to the order Decapoda of crustaceans. Previously, there were no studies regarding gene-specific manipulation in this species. Recently, scientists knocked out the *EcChi4* gene in shrimp using the CRISPR/Cas9 gene editing system and revealed the function of this gene *in vivo*. This is the first time that the microinjection method has been used in *P. carinicauda*, and the mutations were shown to be transmitted to subsequent generations. The use of CRISPR/Cas9 technology in shrimp provides a foundation for knockout or knock-in studies of genes of interest related to their development, growth, metabolism, and reproduction (Gui et al., 2016). This gene editing system will certainly become a robust tool for manipulating the genomes of decapods.

### Amphipoda

#### Parhyale hawaiensis

Homeotic genes (Hox genes) can regulate the specialized biological phenotypes of many arthropod species (Hughes and Kaufman, 2002). Therefore, scientists are committed to studying how Hox proteins lead to morphological diversity (Pearson et al., 2005). It has been noted that Hox genes play a role in generating limb diversity (Jung et al., 2014), but the precise functions of these genes have rarely been studied in crustaceans. Martin et al. combined CRISPR/Cas9 and RNAi technology to analyze six Hox genes in *Parhyale hawaiensis* (Crustacean, Amphipoda). They targeted the six Hox genes *Dfd, Antp, Abd-A, Abd-B, Scr*, and *Ubx*, and injected Cas9/sgRNA mixtures into one-cell embryos of *P. hawaiensis*. The results revealed the function of these six genes in different appendage regions of *P. hawaiensis*, and they also indicated the different phenotypic effects caused by deficiencies in different Hox genes (Martin et al., 2016).

### Cladocera

#### Daphnia magna

*Daphnia magna* (Crustacea, Cladocera) is a member of the zooplankton found in fresh water. Because they are easy to culture are parthenogenetic clones and have a short generation time, they are attractive for scientific research (Hebert, 1978). Interestingly, *D. magna* is able to detect ecotoxicity and environment status through its feeding behaviors (Zhu et al., 2008). A cDNA library was constructed, and the expressed sequence tags of *D. magna* were determined (Watanabe et al., 2005). Scientists utilized the published genetic information to analyze the eye phenotype gene *eyeless* by injecting Cas9/sgRNA mixtures into eggs and obtained mutants with defects in the *eyeless* gene accompanied with a deformed eye phenotype (Nakanishi et al., 2014). This is the first example of genetic modification in *D. magna*; therefore, the experimental results may be of great value to future studies in this species.

## sgRNA design and off-target effects

SgRNAs directly determine the editing efficiency of CRISPR/Cas9, and the design of sgRNAs relies on high-quality genomic data and complete gene annotations (Mohr et al., 2016). The classical sgRNA-Cas9 structure consists of exactly 20 base pairs followed by an NGG (PAM) site; additionally, there are several non-canonical PAMs used for CRISPR/Cas9-mediated DNA cleavage, and reports have indicated that NGA PAMs have a relatively higher cleavage efficiency in human cells (Zhang Y. et al., 2014). Some studies have indicated that the initial base of an sgRNA affects the cleavage efficiency, and different promoters will drive different base-initiated sgRNAs. The U6 promoter can drive sgRNAs that require a G at the start of the protospacer in humans (Ding et al., 2013). Ranganathan et al. indicated that H1 promoter-expressed sgRNAs can be used to target all AN_19_NGG and GN_19_NGG genome sites in humans and other eukaryotic species (Ranganathan et al., 2014). Zeng et al. showed that sgRNAs with various types of N_20_NGG sequences were more suitable and useful for genome editing than GN_19_NGG sgRNAs, and they indicated U6-driven sgRNAs beginning with four different nucleotides could cause single site mutations in the *B. mori* genome (Zeng et al., 2016). A genome-wide sgRNA library was constructed to aid the screening and study of functional genes in *Drosophila* (Bassett et al., 2015). Moreover, a study demonstrated that an sgRNA paired with 10–12 bp close to the PAM site is crucial for identifying target sites; accordingly, mismatches 8–10 bp away from the PAM site may not have a significant effect on target site recognition (Jiang et al., 2013). Scientists also showed that the 20 nt sgRNA target sequence could be shortened to 17 nt and combined with a double-nicking strategy, which significantly reduced off-target effects (Ran et al., 2013; Fu et al., 2014). It has been noted that the optimal sgRNA length is 18–19 nucleotides (Kunzelmann et al., 2016). In the CRISPR/Cpf1 system, the Cpf1 enzyme is smaller than the standard Cas9 and easily delivered into cells and tissues; these advantages allow it to more efficiently edit the genomes of different organisms (Zetsche et al., 2015).

Several technologies have been derived from the CRISPR/Cas9 system, including CRISPR activation (CRISPRa) technology can catalyze inactivation of Cas9 (dCas9) coupled with transcriptional activators to promote the transcription of specific gene (Gilbert et al., 2013). CRISPR interference (CRISPRi) technology is a CRISPR-associated catalytically inactive dCas9 protein to silence the expression of endogenous gene by coupling of dCas9 to a transcriptional repressor domain and (Choudhary et al., 2015). CRISPR-on technology is a two-component transcriptional activator consisting of a nuclease-dead Cas9 (dCas9) protein fused with a transcriptional activation domain and sgRNAs with complementary sequence to gene promoters (Cheng et al., 2013) and co-CRISPR technology can significantly improves the screening efficiency in identification of genome editing events (Kane et al., 2016). Different CRISPR systems have different sgRNA design guidelines. In the CRISPR/Cas9 system, the target region should be gene-specific and usually in an exon. In the CRISPRa system, the target region should be 50–500 bp upstream of the TSS site, while the target region of the CRISPRi system should be near the transcription start site (TSS) site; most important in all of these systems is to avoid off-target effects as much as possible (Mohr et al., 2016). Since CRISPR/Cas9 technology is used to manipulate the genomes of diverse organisms, preventing off-target effects is a challenging issue. Ren et al. analyzed off-target efficiency and established a Web-based resource that is helpful for genome editing in flies (Ren et al., 2013). An sgRNA scoring algorithm was designed to produce highly efficient sgRNAs (Moreno-Mateos et al., 2015). A previous report showed that Cas9s from *S. pyogenes, Streptococcus thermophiles*, and *Neisseria meningitidis*, abbreviated as SpCas9, StCas9, and NmCas9, could all direct an sgRNA to cleave a target sequence (Hou et al., 2013). To improve the efficiency of genome editing, researchers found two smaller Cas9s (St1Cas9 and SaCas9) derived from *S. thermophilus* and *Staphylococcus aureus*, respectively, which are homologous to SpCas9. In addition, SpCas9 can recognize a transformed PAM site, such as NGCG, which significantly reduces the off-target effect (Gao et al., 2015; Kleinstiver et al., 2015).

## Discussion

The CRISPR/Cas system was originally derived from an adaptive immune defense mechanism of bacterial and archaeal species that is used to deal with viruses or conjugative plasmids (Richter et al., 2013). Many researchers are interested in this natural mechanism and have used it in many organisms. In 2013, genomic editing technology of entomology field is kicked off since Gratz et al. applied CRISPR/Cas9 system to manipulate the genome in *D. melanogaster* (Wang et al., 2013), whereafter, CRISPR/Cas9 technology is widely used in many insects and non-insect arthropods. However, the editing efficiency is lower than that of mammals and we summarized the possible reasons: the first is insect eggs are smaller and fragile which will damage in injection process; secondly, the plasmid DNA serves as source of Cas9 and sgRNA; thirdly, the culture conditions after injection will affect hatchability. In 2013, Bassett et al. showed that injection of RNA into the *Drosophila* embryo can induce highly efficient mutagenesis of desired target genes in up to 88% of injected flies via CRISPR/Cas9 system (Bassett et al., 2013). According to the above method, Wang et al. manipulated the *HaCad* gene in *H. armigera* by injecting RNA into eggs (Wang J. et al., 2016) and Li et al. edited the genome of *N. vitripennis* by injecting sgRNA and Cas9 protein (Li et al., 2017), which greatly increased the efficiency of gene editing and the experiment process became more easier. Although the efficiency of genome editing is increased, the RNA is still not as stable as plasmid, which needs to be further resolved.

In this review, we summarized the use of the CRISPR/Cas9 technique in insect and non-insect arthropods, including Lepidoptera, Coleoptera, Orthoptera, Diptera, Acarina and Decapoda. In this paper, we prepared a comprehensive summary. Compared with other widely used genome editing techniques, such as ZFN and TALEN, the CRISPR/Cas9 system is simple and easy to use, affordable, and suitable for not only model organisms but also unusual species. We also described the mechanism and application of two new CRISPR systems, CRISPR/Cpf1 and CRISPR/C2c2. In arthropods, CRISPR/Cas9 technology can be used to explain resistance mechanisms that may cross species boundaries, and the technology has also provided several ideal strategies for pest regulation (Awata et al., 2015; Douris et al., 2016; Koutroumpa et al., 2016; Li Y. et al., 2016; Wang J. et al., 2016; Khan et al., 2017). Moreover, the CRISPR/Cas9 system has also been used to verify functional genes associated with insect physiological processes, such as embryonic development and abdominal segment determination (Bi et al., 2016; Huang et al., 2016; Li X. Y. et al., 2016; Liu H. et al., 2017), female sex pheromone perception (Zhu et al., 2016), sex determination and life span (Xu et al., 2017; Zhang Z. et al., 2017), pigmentation pattern formation (Khan et al., 2017), eyespot formation and development in butterflies (Perry et al., 2016; Zhang and Reed, 2016), and migration behavior (Markert et al., 2016). Meanwhile, insect resistance has also been analyzed using CRISPR/Cas9 genome manipulation tools (Douris et al., 2016; Wang J. et al., 2016; Zimmer et al., 2016; Bajda et al., 2017; Ngai and McDowell, 2017). Furthermore, the aim of some of the reviewed studies was to validate CRISPR systems by knocking out genes that have a visible mutant phenotype (Wang et al., 2013; Liu Y. et al., 2014; Abudayyeh et al., 2016; Kim H. et al., 2017). In addition, researches achieved the prevention and treatment of a human infectious disease and produced live attenuated malaria vaccines using CRISPR/Cas9 technology (Singer and Frischknecht, 2017).

## Prospects

The evolution of genome editing technology from the first generation ZFNs to the second generation TALENs and now the third generation CRISPR/Cas9 is regarded as a historical leap. The CRISPR/Cas9 system was honored as one of the top ten scientific breakthroughs by Science Magazine in 2013. It has many important applications; for example, CRISPR/Cas9 technology has the potential to promote cancer immunotherapy (Eyquem et al., 2017), to treat eye diseases (Kim E. et al., 2017; Kim K. et al., 2017), to breed transgenic pigs (Burkard et al., 2017), and to cultivate disease-resistant cattle (Gao et al., 2017). The Zhang laboratory has proposed a new CRISPR/Cpf1 system, known as the type II CRISPR/Cas system (Jinek et al., 2012); this system is simpler and more convenient than CRISPR/Cas9 and requires only a single crRNA in an activated complex with Cpf1 (Cas12a) to perform multiplexed genome editing (Zetsche et al., 2017). Unlike Cas9, Cpf1 cleaves target DNA near 5′ T-rich PAM (TTN) sites and leaves a short overhang end, which is more conducive to the precise insertion of a DNA sequence (Zetsche et al., 2015). This new system has been applied in many areas; recently, Lei et al. showed that on the target strand, Cpf1 cleaved at approximately the 22nd base relative to the PAM site, while cleavage on the non-target strand was affected by the spacer length (Lei et al., 2017). Kim et al. indicated that the CRISPR/Cpf1 system improve the fat content of soybeans (Kim H. et al., 2017). Port et al. used the CRISPR/Cpf1 system in *Drosophila* and demonstrated that a tRNA-sgRNA system can promote genome editing (Port and Bullock, 2016). Later, Zhang's group discovered the CRISPR effector C2c2 (Cas13a), which enable the editing of ssRNA target sequences. This discovery suggested that the CRISPR/C2c2 system could be used to treat several RNA viruses (Abudayyeh et al., 2016). Recently, an RNA-targeted CRISPR system was found; in this system, a new Cas enzyme (Cas13b) was identified, and compared with Cas13a, it is more suitable for fine-tuning gene function (Smargon et al., 2017). Additionally, CRISPR interference (CRISPRi) technology provides a superior tool for completely repressing the expression of target DNA in mycobacteria (Choudhary et al., 2015). These emerging systems suggest that in the future, gene editing will become even more efficient and convenient. The room for development in arthropods, especially insect species, is extensive, and we look forward to more impressive achievements.

## Author contributions

DS and ZG were both the primary authors who were responsible for the design and writing of the manuscript. YL and YZ assisted with writing and editing the manuscript. All authors listed, have made substantial, direct, and intellectual contribution to the work, and approved it for publication.

### Conflict of interest statement

The authors declare that the research was conducted in the absence of any commercial or financial relationships that could be construed as a potential conflict of interest.
